# ATP5D Is a Potential Biomarker for Male Fertility

**DOI:** 10.1155/2023/4923614

**Published:** 2023-01-11

**Authors:** Yi-Bo Wang, Chen-Yang Zhai, Guan-Ping Yao, Ting-Bao Chen, Lu-Lu Xue, Lin Zhou, Liu-Lin Xiong, Ting-Hua Wang

**Affiliations:** ^1^School of Basic Medical Sciences, Jinzhou Medical University, Jinzhou, Liaoning Province 121001, China; ^2^Institute of Neuroscience, Kunming Medical University, Kunming, Yunnan Province 650031, China; ^3^Reproductive Center, Affiliated Hospital of Zunyi Medical University, Zunyi, 550000 Guizhou, China; ^4^Animal Zoology Department, Kunming Medical University, Kunming, Yunnan Province 650031, China; ^5^State Key Laboratory of Biotherapy, Sichuan University, Chengdu, 610041 Sichuan, China; ^6^Department of Anesthesiology, Affiliated Hospital of Zunyi Medical University, Zunyi, 550000 Guizhou, China

## Abstract

**Background:**

Infertility is a global medical and social problem that affects human health and social development. At present, about 15% of couples of the right age in the world are infertile. As all we know, genetic defects are the most likely underlying cause of the pathology. *ATP5D* is also known as the delta subunit of mitochondrial ATP synthase. Mitochondria maintain sperm vitality, capacitation, acrosome reaction, and DNA integrity through ATP. Mitochondrial damage can trigger energy synthesis disorders, resulting in decreased sperm quality and function or even disappearance. The specific role of *ATP5D* in regulation of the male reproductive system remains elusive.

**Methods:**

In this study, semen from normal and infertile males were collected and their indicators were examined by analysis of routine sperm parameters; *ATP5D* protein content in semen was examined by ELISA. Singer sequencing was used to detect whether there was a mutated of *ATP5D* in semen. Meanwhile, *ATP5D* knockout (KO) and knockin (KI) male mice were selected at 8-12 weeks of age and mated with adult wild-type (WT) female mice for more than two months to assess their fertility and reproductive ability. Morphological changes in tissues such as testes and epididymis were observed by HE staining; spermatozoa were taken from the epididymis of the mice; sperm counts were performed and morphological changes were observed by Diff-Quik staining.

**Results:**

The results showed that the expression of *ATP5D* in infertile males was significantly lower than that in normal males (*P* < 0.001) and the normal morphology rate of spermatozoa was much lower than that of normal males, and the sequencing results showed no mutations. The animal reproductive experiments showed no significant changes in the number of fertility in KO/KI mice compared with WT mice, but the duration of fertility was significantly longer (*P* = 0.02). The testicular cells in KO mice were loosely arranged and disorganized, the lumen was larger, the interstitial cells were atrophied, and the number of spermatozoa was reduced and the malformation rate was higher in WT males. This suggests that *ATP5D* is an essential protein for sperm formation and fertility in male mice and may be used as a biomarker of male fertility.

**Conclusion:**

This study found *ATP5D* correlated with male infertility and the expression levels were significantly reduced in the seminal plasma of all male infertile patients without gene mutations. KO male significantly prolonged fertility time and impaired testicular histomorphology. This suggests that *ATP5D* may be associated with spermatogenic function and fertility in male mice and may be used as a biomarker for male fertility. Future studies are required to elucidate the potential mechanisms. The trial registration number is KLL-2021-266.

## 1. Introduction

Male infertility is clinically defined as infertility caused by male factors. According to the definition of the World Health Organization (WHO), infertility is that a men and a women have normal sexual intercourse after marriage, do not use contraceptive, and live together for one year without pregnancy [1]. Due to increasing pressure, poor living habits, and polluted living environment, the incidence of this disease is currently on the rise, which greatly affects the physical and mental health of patients and reduces the quality of life. Currently, it has become a global health problem [2]. The quality of male sperm has decreased by about 50% in the last half century. Infertility has become the third most difficult disease in the world after cardiovascular disease and tumor, which seriously threatens human reproductive health [3, 4]. Therefore, the study of male reproductive health are of very important physiological and social significance. The pathogenesis of male infertility is complex and can be caused by congenital or secondary urogenital system abnormalities, gonad infections, endocrine disorders, varicocele, mitochondrial dysfunction, or immune disorders. About 40% of patients suffer from idiopathic infertility. Its molecular mechanism is not fully understood [5]. According to the latest epidemiological data at home and abroad, about 15% couples of appropriate age are currently infertile [6, 7] and genetic defects are the most likely underlying cause of the pathology [8, 9]. With the development of molecular biology techniques, exploration of male infertility caused by genetic etiology has noticeably attracted scholars' attention. In recent years, a variety of genes were found to be implicated in male infertility [10, 11]. Although many genes for male infertility have been identified, the genetic causes of most male infertility are currently uncharactersitic [8–10]. The current diagnosis is mainly based on routine and biochemical indices of semen [12–14]. However, these tests lack high diagnostic specificity and sensitivity. About 30%-50% of male patients with idiopathic infertility have completely normal indicators [15]. It is well known that sperm production, development, and fertilization are affected by many factors. Abnormalities in any link may lead to infertility. Therefore, the above indicators alone cannot accurately reflect the internal structure and function of sperms, which make it impossible to comprehensively assess male fertility [16, 17]. Therefore, it is still necessary to explore new noninvasive diagnostic markers for male infertility.

*δ*-Subunit of the catalytic core of mitochondrial adenosine triphosphate synthase (*ATP5D*) is a constitutive protein of mitochondria, also known as the delta subunit of mitochondrial *ATP* synthase. It consists of a hydrophilic head of F1 protruding outside the membrane and a hydrophobic tail of F0 embedded in the membrane. It plays an important role in transporting coupled protons and *ATP* production [18, 19]. Downregulation of *ATP5D* expression results in a decrease in the level of adenosine triphosphate synthase [20, 21], which affects sperm vitality. Mitochondria are one of the most important organelles in cells, which are involved in spermatogenesis, capacitation, sperm vitality, and fertilization. The main functions include participation in energy conversion, tricarboxylic acid cycle, oxidative phosphorylation, and storage of Ca^2+^. Mitochondria are the key to metabolism and bioenergy conversion. If mitochondria get dysfunctional, sperm synthesis will often be hindered [22]. At the same time, studies have shown that sperm mitochondrial membrane potential is positively correlated with sperm vitality and sperm fertilization rate [23, 24]. Increased *ATP5D* expression may be a marker for increased metabolic activity, thereby increasing oxidative stress in embryos [25, 26]. In clinical studies, the expression of *ATP5D* significantly increased and the mean of sperm vitality increased in patients after varicocelectomy [27], suggesting that mitochondrial protein deficiency played an important role in varicocele [28]. Meanwhile, preoperative sperm vitality was lower possibly because of the low expression of *ATP5D* [29, 30]. In recent years, researches on andrology, especially for the testis, have obtained a lot of data, but the clinical researches on male infertility have made little progress and limited impact on clinical treatment. Causes for the deterioration of semen quality and male fertility have become the focus on male reproductive health. They are in the urgent need of attention.

Based on this, the study intended to use the semen from male patients with infertility and males with normal reproduction to evaluate the main indicators, the semen morphology, and the difference in *ATP5D* expression level. The effects of *ATP5D* on male infertility were studied. At the same time, we analyzed the correlation between the differences in *ATP5D* expression level and indicators of semen to find important indicators. Whole-genome DNA was extracted from semen samples. The full-length sequence of the *ATP5D* gene was amplified by polymerase chain reaction (PCR). The amplified products were sequenced by Sanger. The bioinformatic analysis was performed to compare whether the changes of gene mutation sites had an effect on reproductive function. Subsequently, 8-12-week-old *ATP5D* knockout (KO), knockin (KI), and wild type (WT) mice were divided into groups to conduct fertility experiments that aimed to observe the effects of *ATP5D* on the fertility of mice and to provide experimental guidance for clinical treatment for male infertility.

## 2. Materials and Methods

### 2.1. Study Population

The semen samples were taken from 65 males with normal reproduction function and 85 male patients with infertility. All the males, aged within 20-45 years old, sought treatment in the Affiliated Hospital of Zunyi Medical University from June to August in 2021. Patients were fully informed to obtain informed consent from patients before semen specimen collection, which was approved by the Ethics Committee of the Affiliated Hospital of Zunyi Medical University (ethical review approval no. KLL-2021-266). For the normal group, who had a history of causing pregnancy, underwent prenatal examinations. All the indicators of the semen examinations were normal. Specific data were listed as follow: sperm volume ≥ 1.5 mL, pH ≥ 7.2, sperm concentration ≥ 15 × 10^6^/mL, PR ≥ 32%, and normal morphological rate ≥ 4%. For the infertile group, (1) they met the diagnostic criteria which were published in *Laboratory Manual for the Examination and Processing of Human Semen* by WHO; (2) their wives had no infertility disease; they were married and cohabited for more than one year; and (3) they had a good sexual relationship and did not take any contraceptive measures after marriage. Exclusion criteria were listed as follows: (1) infertility was caused by female factors; (2) they took medicine that affected semen quality over the past 3 months; (3)patients suffered from orchitis, epididymitis, and purulent semen with white blood cells (WBCs) in semens > 1.0 × 10^6^/mL; (4) infertility caused by infections in urogenital system and other factors; (5) patients with severe varicocele, cryptorchidism and chromosomal abnormality; (6) patients with malignant tumors; and (7) patients with severe systemic disease. Samples were collected and analyzed in accordance with the standards of WHO (World Health Organization). After the semen became liquid, semen underwent a routine analysis using a fully automated computer-assisted semen analysis system (CASA) as suggested in the literature [31]. The semen volume, pH, sperm morphology, sperm concentration, and sperm vitality from the infertile patients were observed. According to *Laboratory Manual for the Examination and Processing of Human Semen*, the data were listed as follows: semen volumes ≥ 1.5 mL, pH ≥ 7.2, sperm morphology ≥ 4%, total sperm counts ≥ 39 × 10^6^, sperm densities ≥ 15 × 10^6^/mL, forward movement ≥ 32%, and sperm vitality > 60%. If one or more of the indicators were lower than the above criteria, the semen would be considered abnormal.

### 2.2. Experimental Animals

The animal protocol for this study has been approved by the Animal Care and Welfare Committee of Kunming Medical University. The C57BL/6 mice used in this experiment were bred in the Experimental Animal Center of Kunming Medical University and were raised separately. The mice were housed under a 12-hour light-dark cycle with food and water provided ad libitum. Constant temperature (20-22°C) and humiditiy (50%-70%) were maintained. *ATP5D* KO and KI rats were constructed in Cyagen Biosciences (Cyagen, Guangzhou, China). All the experiments were performed in accordance with The Guide for Care and Use of Laboratory Animals published by the National Institutes of Health.

### 2.3. Diff-Quik Staining

Sperm morphology was stained with Diff-Quik (Solarbio, Beijing). By this method [32], the acrosome regions of the sperms were stained lavender. The nuclear regions were stained violet blue. The middle and main segments were stained light red. It was a simple and efficient method. Two hundred sperms were counted in strict accordance with the standard of sperm morphology. The percentage of normal sperm morphology was obtained. The percentage of ≥4% was considered as normal.

### 2.4. Enzyme-Linked Immunosorbent Assay (ELISA)

The *ATP5D* levels in the semen from different groups were detected to explore the effect of *ATP5D* expression on male infertility. According to the manufacturer's protocol, an enzyme-linked immunosorbent assay (ELISA) kit (Jiangsu Meimian Industrial Co., Ltd.) was used. About 100 *μ*L of samples or standard products was added to the wells and then incubated at 37°C for 2 hours (h). After removing the liquid, 100 *μ*L of biotin conjugates was added to each well and incubated at 37°C for 1 h. After cleaning for 3 times with washing buffer, the products were incubated with 100 *μ*L of streptavidin-horseradish peroxidase for 1 h at 37°C. 100 *μ*L of 3.30,5.50-tetramethylbenzidine was added to each well for colour development. After the reaction had stopped, the absorbance was measured by using a microplate reader (Thermo Fisher Scientific, MK-3) which was set to 450 nm. The concentration of ATP5D in each sample was calculated by using the standard curve.

### 2.5. Singer Sequencing

The semen samples from the normal group and the infertile group were sent to Sangon Bioengineering (Shanghai) Co., Ltd., for sequencing. The sequence alignment was analyzed in National Center for Biotechnology Information (NCBI). Genomic DNA was extracted from the semen by using a genomic DNA extraction kit Sangon Bioengineering (Shanghai Co., Ltd., batch number: B518264). There were five exon regions in total, which were divided into two sequence fragments to design specific primers. One of the sequences was 1063 bp in length. The forward primer was *ATP5D*-1&2-F (5′-AAACTGCAACCCCCAGAATAC-3′) and the reverse primer was *ATP5D-*1&2-R (5′-CTATTTTTTCTCGCAGCCTCAAC-3′). The other sequence was 1024 bp in length. The forward primer was *ATP5D*-3&4&5-F (5′-GTTTGCACACTCAGGACACAGAC-3′) and the reverse primer was *ATP5D*-3&4&5-R (5′-CTCGAGCCTGCTTCCTACGG-3′). Parameter setting for the PCR amplification system was listed as follows: 25 *μ*L, predenaturation at 95°C for 5 minutes (min), denaturation at 94°C for 30 seconds (s), annealing at 63°C for 30 s, and extension at 72°C for 30 s for 30 cycles and extension at 72°C for 10 min. 5 *μ*L of PCR products was taken and 1% agarose gel electrophoresis was performed. After confirming that the band size was as expected, sequence alignment was analyzed in NCBI.

### 2.6. Genotype Identification of ATP5D KO and KI Rats

For the rats at 7–10 days after birth, the toes and tail tips were collected and numbered. Then, rats' genomic DNA was extracted by using TransGen's genomic DNA extraction kit (ee101-12), and PCR detection was performed with the amplification primer: mouse ATP5D KO-F: 5′-CTGATGTGCTCAGGGTGTTGACC-3′; mouse ATP5D KO-R: 5′-GCTGCTGATGTTAGGGAGGAGGC-3′. Mouse ATP5D KI-F: 5′-CTGATGTGCTCAGGGTGTTGACC-3′; mouse ATP5D KI-R: 5′-GC TGCTGATGTTAGGGAGGAGGC-3′.

### 2.7. Assay of Reproductive Ability

The fertility test was performed by mating 8-12-week-old KO or KI male with WT female mouse. According to the following scheme: (1) KO male mice+WT female mice; (2) KO male mice+KI female mice; (3) KI male mice+WT female mice; (4) KI male mice+KO female mice; and (5) WT male mice+WT female mice (*n* = 5). Each cage included a male mouse and a female mouse. The housing environment was kept in a 12-hour light-dark cycle with food and water provided ad libitum. The temperature was maintained within 22-26°C. The mating experiment was repeated for 8 weeks for each male mouse. All females were monitored during pregnancy. Dates of birth and the number of pups were recorded for all litters.

### 2.8. Tissue Harvest

Anesthesia was induced by inhalation of 5% isoflurane and maintained at 3%. The mice were immobilized in the supine position. The abdominal skin was lifted. The abdominal cavities were opened at the xiphoid process to expose the muscle layers. The thoracic cavities were cut along the costal arches on both sides. The xiphoid process was turned up with hemostatic forceps to expose the thoracic cavities. The connective tissues around the heart were cut to separate the pericardium, dissociate the heart, and fully expose the heart and the ascending aorta. A perfusion needle was punctured from the apex of the heart and went deeper into the aortic root to connect an extension tube. The needle was fixed with an arterial clip. The right atrial appendage was cut. About 50 mL of 0.9% sodium chloride was rapidly perfused. After the liquid flowing out of the right atrial appendage had become clear, about 50 mL of 4% paraformaldehyde was slowly perfused. It could be seen that the mouse limbs were stretched and twitched. The tail got stiff and the whole body became rigid. Their right testes were taken for PCR. The epididymis was used for sperm counting and staining. The left testes and epididymides were placed in 4% paraformaldehyde solution for HE staining after 24 h of fixation.

### 2.9. HE Staining

To observe the morphology of the testicular seminiferous tubules and the morphological differences between the testes and epididymides, HE staining was performed (C0105; Beyotime Institute of Biotechnology). The tissues were fixed in 4% paraformaldehyde solution for more than 24 h. Then, they were dehydrated in graded alcohol and embedded in paraffin to be made into coronal sections (3 *μ*m). The sections were immersed in hematoxylin for 3-5 min and cleaned with tap water for 3 min. Then, they were differentiated with hydrochloric acid and alcohol and cleaned again with running water for 3 min. Next, the sections were immersed in 95% alcohol and were stained with eosin for 1-2 min. Afterwards, they were dehydrated in a gradient of alcohol and made transparent in xylene. After drying naturally, the sections were sliced in neutral resins. The morphology of the sections was observed under a microscope.

### 2.10. Analyses of Mouse Sperm Counting

The mice's epididymides were taken and put it into a culture dish containing 1 mL PBS buffer at 32°C. Then, tissue scissors were used to cut several small openings on the tail of the epididymides to allow the sperms to move out. The dish was placed in an incubator at 32°C for 10 min to allow the sperms to move from the tails of the epididymides into the phosphate-buffered saline (PBS) buffer. The PBS buffer, mixed with epididymal sperm, was carefully and slowly mixed by pipetting and transferred to a sterile EP tube. Then, the mixture was centrifuged at 2000 rpm at 4°C for 5 min. Part of the supernatant was removed. 2% formalin solution was added at room temperature. Next, the epididymal sperms were fixed for 10 min. Afterwards, they were diluted in PBS. Finally, 10 *μ*L of the mixture was applied onto a hemocytometer to count cell numbers under an optical microscope.

### 2.11. Mouse Sperm Staining

10 *μ*L of semen samples were taken for sperm smear. After fixation, the samples were stained with Diff-Quik. All the steps were performed according to the instructions of the kit (Solarbio, Beijing). Under the microscope (DM400B, Leica), 200 sperms were counted in strict accordance with the sperm morphology standard. The percentage of sperms with normal morphology was obtained. The percentage of ≥4% was considered as normal.

### 2.12. Statistical Analysis

The samples were obtained from at least 5 mice for the animal experiment (*n* = 5). All the experiments were independently repeated at least three times. All statistical analyses were done by using SPSS 21 (IBM Corporation, NY, USA). Data were expressed as mean ± standard deviation (SD). The comparison between two groups was performed by using independent sample *t*-test. For comparison of multiple groups, ANOVA with least-significant difference (LSD) or Dunnett's T3 post hoc test was applied; if equal variances were found, LSD was performed; otherwise, Dunnett's T3 was used. Clinical data were analyzed based on quantitative and qualitative data. Receiver operating characteristic (ROC) curves were drawn to evaluate the diagnostic value of *ATD5D* for male infertility. The correlation between *ATP5D* levels and various indicators of semen analysis was analyzed by Pearson linear correlation analysis. *P* < 0.05 was considered statistically significant.

### 2.13. Ethics Approval and Consent to Participate

Both trials were ethically approved by the Affiliated Hospital of Zunyi Medical University (approval number: KLL-2021-266).

All the participants were fully informed about the collection and the informed consent was obtained from them.

### 2.14. Role of the Funding Source

The funder of the study had no role in study design, data collection, data analysis, data interpretation, or writing of the report. The authors had full access to all the data in the study and accept responsibility to submit for publication.

## 3. Results

### 3.1. Multiple Defects Are Observed in Spermatozoa from Infertile Male

**Figure 1:**
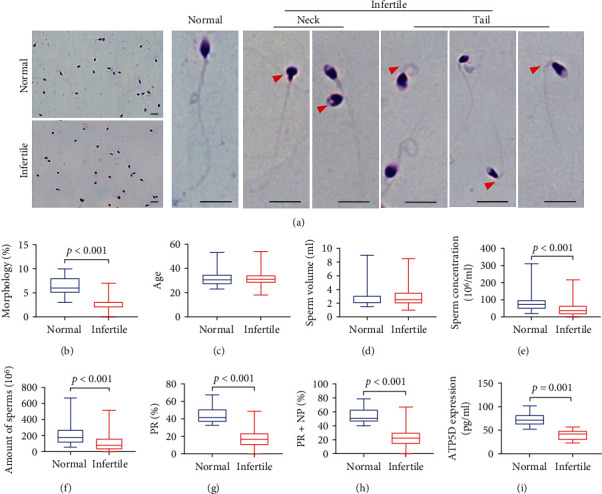
Multiple defects are observed in spermatozoa from infertile male. (a) The morphology of the spermatozoa from a fertile normal and an infertile group under light microscopy. Multiple images were taken, and typical features of abnormal spermatozoa are exemplified, such as abnormal head and mitochondrial sheath and the short or absent flagella. Scale bar: 20 *μ*m. (b) Male sperm normal morphology rate. (c–h) The results of age, ejaculation volumes, sperm counts, sperm concentrations, total vitality, and forward vitality of the normal group and the infertile group, respectively. (i) The differences in ATP5D protein expression between normal and infertile male.

Diff-Quik staining revealed that the spermatozoa from the normal group displayed normal morphology. That the sperm heads from the normal group were smooth and regular. Most of them were oval and the colour was dark purple. The acrosomes were shown clearly and the colour was light purple. The middle sections were thin. The colour of both the main axes and the long axes of the heads were light red. However, as indicated by the red arrows, the spermatozoa from the sterility group showed multiple morphological abnormalities, including abnormal head morphology, such as round, flat head, and head insertion asymmetry. The sperm tails showed abnormalities including missing or significantly abnormal mitochondrial sheaths and abnormal flagella (Figure 1(a)). The abnormality rate of sperm in infertile group was significantly higher than that in normal group (Figure 1(b), *P* < 0.001), Subsequently, the sperm samples were then routinely analyzed by an automatic sperm analyzer and the detail could be seen in (Table 1). We performed statistical analysis on important indicators including age, semen volumes, sperm counts, sperm concentrations, total vitality, and forward vitality. The results showed that there was no statistical significance in the age and ejaculation volume (Figures 1(c) and 1(d)*P* > 0.05). However, the sperm counts, sperm concentrations, PR+NP, and PR were higher than those in the infertile group; the difference was statistically significant (Figures 1(e)–1(h), *P* < 0.001). To study the effect of *ATP5D* on male fertility, ELISA was used to detect the expression level of *ATP5D* protein in samples from the two groups. Compared with the normal group, the expression of *ATP5D* in the infertile group was significantly lower, the difference was statistically significant (Figure 1(i), *P* <0.001).

### 3.2. *ATP5D* Protein Expression Correlates with Sperm Routine Parameters

**Figure 2:**
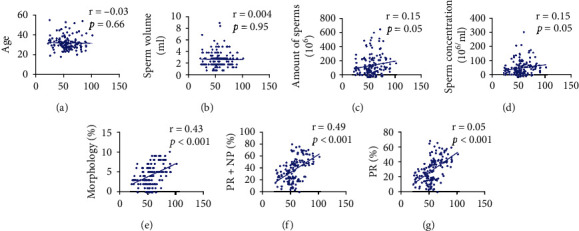
Correlation analysis of routine indicators of sperms and *ATP5D* levels.

In order to verify whether the routine indicators of sperms were correlated with *ATP5D*, we performed a correlation analysis. The results showed that the *ATP5D* levels in sperms were weakly correlated with age and ejaculation volumes (Figures 2(a) and 2(b)) (*r* = −0.03, *r* = −0.004). The *ATP5D* levels in the sperms were positively correlated with total sperm counts, sperm concentrations, normal morphology, forward vitality, and total vitality (Figures 2(c)–2(g)) (*r* = 0.15, *r* = 0.15, *r* = 0.43, *r* = 0.49, *r* = 0.05).

### 3.3. Infertile Men's Semen Was Examined, and no ATP5D Gene Alterations Were Found

Singer sequencing was used to detect whether gene mutation sites existed in the ATP5D of sperms by comparing the sperms from infertile males and males with normal reproductive function. First, the PCR amplification products of the ATP5D genes in the sperms from males were sequenced. A total of 10 samples, including 3 samples from the normal group and 7 samples from the infertile group, were selected. Then, their genomic DNA was extracted for detection. The size of PCR products was detected by electrophoresis (Figure S1A). The determination results of the two fragments were sequenced in NCBI. The results showed that there was no mutation in the sequences. The results are shown in (Figure S1B).

### 3.4. Genotype Identification of Mice

To investigate the function of *ATP5D* in rats with infertility, we established the *ATP5D* KO and KI rats through CRISPR/CAS9 technology. Briefly, two single gRNA (sgRNA) action targets for *ATP5D* gene were designed. Oligonucleotide chains were synthesized according to the sticky end formed by sgRNA vector through BsaI; then, the chains were connected to pRP [CRISPR]-hCas9-U6 carrier after annealing. Construction of sgRNA vector was completed and was confirmed by sequencing (Figure S2A, B). After microinjection for F0, we began to reproduce to acquire Fragment 1 (F1) and verified the genetic modification of *ATP5D* caused by CRISPR/CAS9 via PCR gel electrophoresis to determine WT, homozygote, and heterozygote offspring (Figure S2C, D). WT, KO, and KI were used for the later experiment. The result of qRT-PCR showed that WT mice relative expression of ATP5D was higher than those of KO mice and lower than those of KI mice. (Figure S2E, *P* = 0.01, *P* < 0.001).

### 3.5. Knocking Out *ATP5D* Impacts Fertility in Mice

**Figure 3:**
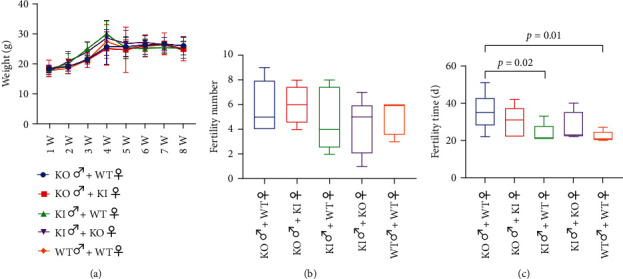
*ATP5D* affects mice's reproduction function. (a) Change of female mice's weights in fertility test. (b) The number of neonatal mice in each group for two months in different cages. (c) The time from caging to giving birth for mice in different cages. Data are presented as mean ± standard deviation.

After obtaining the transgenic mice, we observed the daily behavior and growth status of the mice. Their growth and behaviours were not affected after knockout or overexpression. In order to explore the effect of *ATP5D* on male fertility, we followed the above protocol for fertility testing. The pregnant mice were under observation every day. Mice were weighed daily to determine pregnancy, and daily weight gain was usually observed 13 days after mating (Figure 3(a)). During the fertility test, the number of pups was counted at birth and the time from caging to giving birth were recorded. The results showed that there was no significant difference in number of births between the groups within two months (Figure 3(b), *P* > 0.05). Compared with the WT and the KI mice, the KO mice spent more time in caging and giving birth; the difference had statistical significance (Figure 3(c), *P* = 0.01, *P* = 0.02).

### 3.6. Knocking Out *ATP5D* Impacts Testis Development in Mice

**Figure 4:**
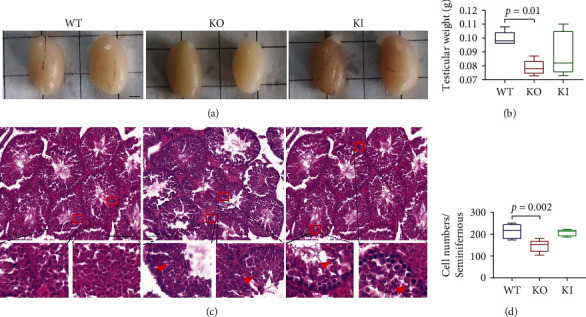
The deficiency of *ATP5D* affects the spermiogenesis and morphology of the testes sections of the WT, KO, and KI male mice after HE staining. (a) Morphology of the testis from the WT, KO, and KI mice. Scale bar = 1 mm. (b) Comparison of the weight of the testis (*n* =5). (c) HE staining results of the testis tissue from the mice. Arrowheads in C indicated the heads of the testis-sperm were severely deformed. Scale bar = 50 *μ*m. (d) Statistical results of the testicular seminiferous tubule cells from the mice (*n* =5).

We isolated the testis tissues from parental male mice after the fertility experiment. It was found that the testis volumes of the KO mice got significantly smaller (Figure 4(a)). The difference in testicular weight loss in KO mice was statistically significant (Figure 4(b), *P* = 0.01). This result suggested that *ATP5D* played an important regulatory role in testicular development. By HE staining, we compared the morphology of the seminiferous tubules and the number of germ cells in seminiferous tubules of mice in each group. We found that the spermatogenic epithelial cells in the testes of WT mice were tightly arranged with clear boundaries, the walls of the spermatogenic tubules were intact, healthy and numerous spermatozoa could be seen in the lumen, and the connective tissue structure between the spermatogenic tubules was intact. However, as indicated by the red arrows (Figure 4(c)), the spermatogenic epithelial cells at all levels in the testes of KO mice were loosely arranged, some interstitial cells disappeared and atrophy occurred, and small vacuoles appeared in the basal area and the lumen of the spermatogenic epithelial cells, probably due to apoptosis. The tissue structure of KI mice became loose, the outer edge was no longer tight, the inner edge was irregularly arranged, the spermatozoa in the lumen became fewer, the number of cell layers in the wall of the tube decreased, and the number of normal form sperm cells in the lumen decreased. The results showed that the number of germ cells in the seminiferous tubules of the testis of the KO mice was significantly less than that of the WT mice (Figure 4(d), *P* = 0.002). Either *ATP5D* KO or KI causes damage to the testicular tissue of male mice. This resulted in the impairment of their fertilization and further affected the development of their offspring.

### 3.7. Knocking Out *ATP5D* Impacts Spermiogenesis and Epididymides Morphology

**Figure 5:**
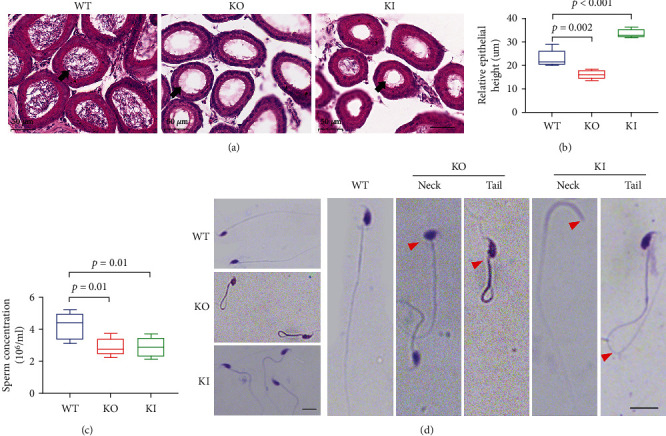
ATP5D affects mice's spermiogenesis and epididymides morphology. (a) Morphology of the epididymis sections of the male mice, arrows in A indicate fewer germ cells. Scale bar: 50 *μ*m. (b) Statistics of thickness of epididymal epithelia. (c) The amount of sperm of the mice. (d) Morphology of the sperm from the KO mice after Diff-Quik staining. Scale bar: 10 *μ*m.

We examined the epididymis of mice by HE staining and found that the epididymal epithelial cells are regularly arranged and the lumen is filled with spermatozoa, but fewer sperm can be seen in the lumen of the KO and KI groups, indicated by black arrow (Figure 5(a)). Compared with WT mice, the thickness of the epididymal epithelia of the KO mice was significantly thinner (Figure 5(b), *P* = 0.002), but KI mice were significantly thickened (Figure 5(b), *P* < 0.001). Subsequently, the number of mature spermatozoa in the tail of the epididymis was counted, and the results showed that the number of epididymal spermatozoa in KO and KI mice was less than that in WT mice, and the difference was statistically significant (Figure 5(c)*P* = 0.002).

Diff-Quik staining demonstrated that mature spermatozoa are present in the epididymis of WT mice. The morphology of mice sperms was different from that of human sperms under microscope. The heads of the mice's sperms were curved and sickle-shaped with smooth surface. The boundary between the acrosome and the posterior acrosome was not clearly shown. The heads and necks of sperms were connected to the ventral side. The necks were slender. The middle segments were consistent in thickness. The main segments were longer, tapered, and had no angled bend.

The KO mice sperm exhibited deformities mainly in the sperms' heads. The deformities included no hook, small head, and no tail. Deformities without heads and tails were common. As for the KI mice, deformities were mainly folding of sperms' heads, breakage of necks, curling of tails, and breakage of tails, which were indicated by the red arrows (Figure 5(d)). These findings further suggest that *ATP5D* deletion or overexpression causes decreased sperm count, poor vitality, and severe sperm abnormalities, which leads to male infertility in male mice.

## 4. Discussion

Male infertility is a common clinical disease that affects the male reproductive system. Any cause of low sperm vitality may trigger male infertility [27]. According to statistics, since 2017, China's birth rate and natural population growth rate have shown a continuous downward trend. In 2018, China's birth rate decreased to 1.094% and the natural growth rate decreased to 0.381%, which were the lowest during 2015-2019. The reduced fertility of couples at childbearing age is one of the important factors leading to the decrease in the birth rate. Moreover, male infertility is the main factor affecting the fertility of couples at childbearing age [33, 34]. As the incidence of male infertility increases, it not only has a negative impact on men's physical health and psychological status, but also terribly affects the relationship between spouses. Low sperm vitality and quality disorders have always been considered as the root causes of male infertility. Exploring an optimal treatment plan to improve semen quality and enhance sperm vitality has become the focus of clinical research.

Mitochondria, as the only organelle in sperm, are powerful. They are located in the middle of the sperms' tails and arranged like a spiral. Various enzyme systems that catalyze and synthesize ATP are stored in the matrix and play a key role in cellular energy metabolism [35]. They take part in biological processes such as calcium signaling, ATP synthesis, mediating cell apoptosis, and production of reactive oxygen species (ROS). Mitochondrial dysfunction is associated with the occurrence of various diseases, such as infertility, aging, and cancer [36, 37]. Mitochondrial defects have been shown to cause pathophysiological changes including male infertility. Mutation or deletion of specific genes can lead to oligospermia and asthenozoospermia [38]. The oxidative phosphorylation reaction in mitochondria can generate enough energy for sperm vitality. At present, the research mechanism of mitochondria involved in male infertility mainly focuses on three aspects: ROS, apoptosis, and Ca^2+^. The production of ROS, which is mediated by mitochondria, is mainly involved in the process of spermatogenesis, differentiation, and maturation, affecting the energy supply of mitochondria and sperm vitality. As a result, oligospermia and asthenozoospermia in men occur. In response to the damage caused by oxidative stress, the clinical application of antioxidant drugs has not achieved the expected effect, but there are still many uncertainties. Little is known about the function of dissociated *ATP5D*, and effects on spermatogenesis and fertility have not been reported in the mammalian reproductive system.

*ATP5D* is located in mice's chromosome 10 and is the *δ* subunit of mitochondrial ATP synthase. This gene encodes a subunit of mitochondrial ATP synthase. Studies have shown that *ATP5D* can also act as a potentially important cell-signaling molecule. *ATP5D* binds to *α*, *β*, *γ*, delta, and epsilon subunits to form the catalytic core (F1) of mitochondrial ATP synthase [39, 40]. Its ability to interact with other subunits within the multiprotein complex suggests that it may also function as a scaffold protein. Mitochondria are one of the most important organelles within the cell that not only play a key role in cellular energy production but also contribute to many processes that are critical to cellular function and dysfunction, including calcium signaling, cell growth and differentiation, cell cycle control, and cell death [41, 42]. Mitochondria are known to be involved in the metabolism of ATP [43]. Therefore, studying the effect of *ATP5D* on male fertility has great significances for improving male sperm quality, fertility, and even human reproduction.

In this study, sperm samples from male patients with infertility and males with normal reproductive function were firstly collected to detect the expression of *ATP5D* and its correlation with male infertility by ELISA. By sequencing analysis, we found that the semen sequence of male infertile patients had no mutation and was consistent with the normal sequence, which indicated that *ATP5D* might affect male fertility at the protein level. Secondly, the role of *ATP5D* in spermatogenesis and the effect on fertility of male mice were investigated by establishing an *ATP5D* transgenic mice model. The sperm counts and sperm vitality of the KO mice decreased significantly, which affected the reproductive function of the mice and caused male infertility. Furthermore, we stained human and mice's sperms with Diff-Quik and observed obvious abnormalities in the structure of the sperms from the KO mice, such as round heads, short tails or no tail, sperm axons, and seminiferous tubules in the testis. Microtubules showed a variety of abnormalities, such as disordered microtubules, microtubules and mitochondrial clusters without a core axofilament structure, or abnormal axon filaments formed by missing central microtubules. At the same time, it was found that the morphology of sperms from the mice was quite different from that of human sperms, which can be summarized as follows: (1) the normal rate of the morphology of the sperms from the mice was very high and the abnormal rate was low, while the abnormal rate of human sperms was high and the normal rate was low. The WHO considers a rate ≥ 4% is normal; (2) the heads of human sperms were oval, and the heads of mice's sperms were curved, sickle-shaped with smooth surface. The boundary between the acrosome and posterior acrosome was not clearly shown. They were obviously smaller than the heads of human sperms; (3) the necks of human sperms were inserted symmetrically, while the necks of mice's sperms were inserted asymmetrically. The heads and necks were connected to the ventral side. The necks were slender and the thickness of the middle sections was the same; and (4) the main part of mice's sperms was inserted. Segments were longer than human sperms and had no angled bends.

Normally, semen is composed of seminal plasma and sperms. Sperms account for about 5% of the semen volume and the rest are seminal plasma. In addition to a large amount of water, fructose, proteins, and polypeptides, seminal plasma also contains a variety of other carbohydrates, enzymes, inorganic salts, and small organic molecules, which can provide nutrition and energy for sperms [44]. Sperms are produced by the testis, matured in the epididymis, and excreted through the vas deferens. Under the influence of carbohydrates and proteins in seminal plasma, sperms gradually mature and acquire vitality and fertilization ability [45]. So far, about 2,000 proteins have been found in seminal plasma [46] and the abnormal expression of some proteins directly affects sperm homeostasis and function, resulting in sperm maturation and vitality disorders [47]. Muhammad Aslam et al. [48] took proteomic methods on high- and low-fertility hybrid bulls. They compared the sperm proteome of high-fertility bulls and low-fertility bulls and verified the sperm proteome by immunoblotting. The results showed that *ATP5D* was highly expressed in high-fertility sperms. It is suggested that the high expression of *ATP5D* in sperms may have a strong correlation with fertility, which is consistent with our findings.

We analyzed the correlation between semen quality and *ATP5D* expression. Age and ejaculation volumes had weaker correlation but had a close correlation with sperm counts, sperm concentrations, proportion of sperms with normal morphology, forward vitality, and total vitality. The result suggested that the expression of *ATP5D* protein was related to male infertility. Studies have shown that the *ATP5D* gene can be used as a candidate marker for identification of idiopathic male infertility [49]. Hosseinifar et al. [30] collected semen samples before and after varicocelectomy. The expression of *ATP5D* significantly increased and the mean value of sperm vitality increased in the patients after varicocelectomy, suggesting that mitochondrial protein deficiency played an important role in varicocele. Increase in the testicular temperature might affect spermatogenesis, sperm vitality, and DNA integrity. Varicocelectomy can improve sperm quality and protein expression. In addition, mitochondria are the core of sperm metabolism, so their normal function is necessary for flagellar vitality, hybrid reaction, and gamete fusion and is associated with decreased sperm quality and infertility [50]. Decrease of sperm vitality may also be triggered by abnormalities in potential of mitochondrial membranes, ROS, mitochondrial Ca^2+^ contents, mitochondrial enzymes and proteome activity, mitochondrial ultrastructure, mtDNA, etc. The results of this study provide further evidence and support for these studies. *ATP5D* is an important subunit of mitochondrial ATP synthase. The expression of *ATP5D* can affect the quality of sperms and the hybridization of sperms and eggs by affecting the synthesis of ATP by mitochondria, thereby affecting male reproductive capacity.

## 5. Conclusion

This study once again demonstrated that *ATP5D* played a crucial role in male fertility and that *ATP5D* deficiency leads to defects in spermatogenesis and sperm function. It is an essential protein for spermatogenesis and fertility in male mice and may serve as a biomarker of male fertility. In conclusion, our findings revealed for the first time the physiological function of *ATP5D* in mice by demonstrating that *ATP5D* is essential for spermatogenesis and male fertility, and that its expression should be maintained at normal levels, and that both elevated or decreased levels affect male fertility rather than genetic mutations. These data strongly suggested an important role of *ATP5D* in human male fertility.

## Figures and Tables

**Table 1:** Sperm routine analysis report of patients.

Characteristics	Normal group (*n* = 65)	Infertile group (*n* = 86)	*P* value
Age (years old)	31.84 ± 6.45	31.6 ± 6.53	0.82
Sperm volume (mL) Amount of sperms (10^6^) Sperm concentration(10^6^/mL)	2.9 ± 1.3	2.82 ± 1.39	0.72
	219.82 ± 141.22	124.15 ± 128.55	<0.001
	82.06 ± 52.39	46.35 ± 42.3	<0.001
Normal morphology (%)	6.3 ± 1.63	2.66 ± 1.83	<0.001
Abnormal morphology (%)	93.66 ± 1.59	97.40 ± 1.56	<0.001
PR+NP (%)	55.46 ± 9.93	22.57 ± 11.37	<0.001
PR (%)	44.18 ± 8.94	16.87 ± 8.8	<0.001
NP	10.28 ± 3.38	5.87 ± 3.7	<0.001
IM	45.53 ± 9.94	76.81 ± 11.63	<0.001
VCL curve rate	45.7 ± 6.55	37.08 ± 8.98	<0.001
VSL linear velocity	20.32 ± 3.65	16.96 ± 4.88	<0.001
VAP average path rate	24.72 ± 3.44	21.92 ± 19.79	0.26
MAD angular displacement	21.14 ± 11.01	25.05 ± 121.18	0.79
ALH side swing amplitude	3 ± 0.47	2.87 ± 3.51	0.77
BCF flagellar frequency	5.08 ± 0.54	4.76 ± 1.16	0.02
LIN linearity	43.75 ± 5.58	42.35 ± 11.31	0.31
WOB Oscillability	53.78 ± 4.68	55.69 ± 50.98	0.76
STR forward orientation	77.91 ± 4.5	74.28 ± 15.17	0.39

## Data Availability

The data that support the findings of this study are available from the corresponding authors upon reasonable request.

## Abbreviations

ATP5D: *δ*-Subunit of the catalytic core of mitochondrial adenosine triphosphate synthase
ATP: Adenosine triphosphate
WT: Wild type
KO: Knockout
KI: Knockin
WBC: White blood cell
CASA: Computer-aided sperm analysis
WHO: World Health Organization
bp: Base pairs
ELISA: Enzyme-linked immunosorbent assay
PCR: Polymerase chain reaction
siRNAs: Short-interfering RNAs
NCBI: National Center for Biotechnology Information
ANOVA: Analysis of variance
HE: Hematoxylin-eosin staining
PBS: Phosphate-buffered saline.

## Supplementary Materials

Supplementary Materials Supplementary Figure 1: singer sequencing for the detection of ATP5D gene mutation sites in male sperm. (A) The size of PCR products was detected by electrophoresis. The detection was repeated twice. The genomic DNA was extracted from 3 samples from the normal group and 7 samples from the infertile group. (B) The comparison of partial sequencing sequence results (no. 1-10 represents 10 samples, respectively; M: marker). Figure 2: design for gene knockout targets and genotyping identification of *ATP5D* transgenic mice. (A, B) Gene knockout target carriers for KO and KI mice, respectively. (C, D) Genotype identification for electrophoresis bands of *ATP5D* KO/KI mice. (E) Verification of the *ATP5D* levels in the testis by PCR. Successful construction of transgenic mice. WT vs. KO, *P* = 0.01; WT vs. KI, *P* < 0.0.1 (*n* = 5). All data are reported as the means ± SD.
