# Supplementary material for: ATP5D Is a Potential Biomarker for Male Fertility
Source: Oxid Med Cell Longev. 2023 Jan 11;2023:4923614. doi: 10.1155/2023/4923614 (PMC9848815; doi:10.1155/2023/4923614)
Supplement: Supplementary Materials — Supplementary Figure 1: singer sequencing for the detection of ATP5D gene mutation sites in male sperm. (A) The size of PCR products was detected by electrophoresis. The detection was repeated twice. The genomic DNA was extracted from 3 samples from the normal group and 7 samples from the infertile group. (B) The comparison of partial sequencing sequence results (no. 1-10 represents 10 samples, respectively; M: marker). Figure 2: design for gene knockout targets and genotyping identification of ATP5D transgenic mice. (A, B) Gene knockout target carriers for KO and KI mice, respectively. (C, D) Genotype identification for electrophoresis bands of ATP5D KO/KI mice. (E) Verification of the ATP5D levels in the testis by PCR. Successful construction of transgenic mice. WT vs. KO, P = 0.01; WT vs. KI, P < 0.0.1 (n = 5). All data are reported as the means ± SD. [file 4923614.f1.docx]

Supplementary Figure 1

**
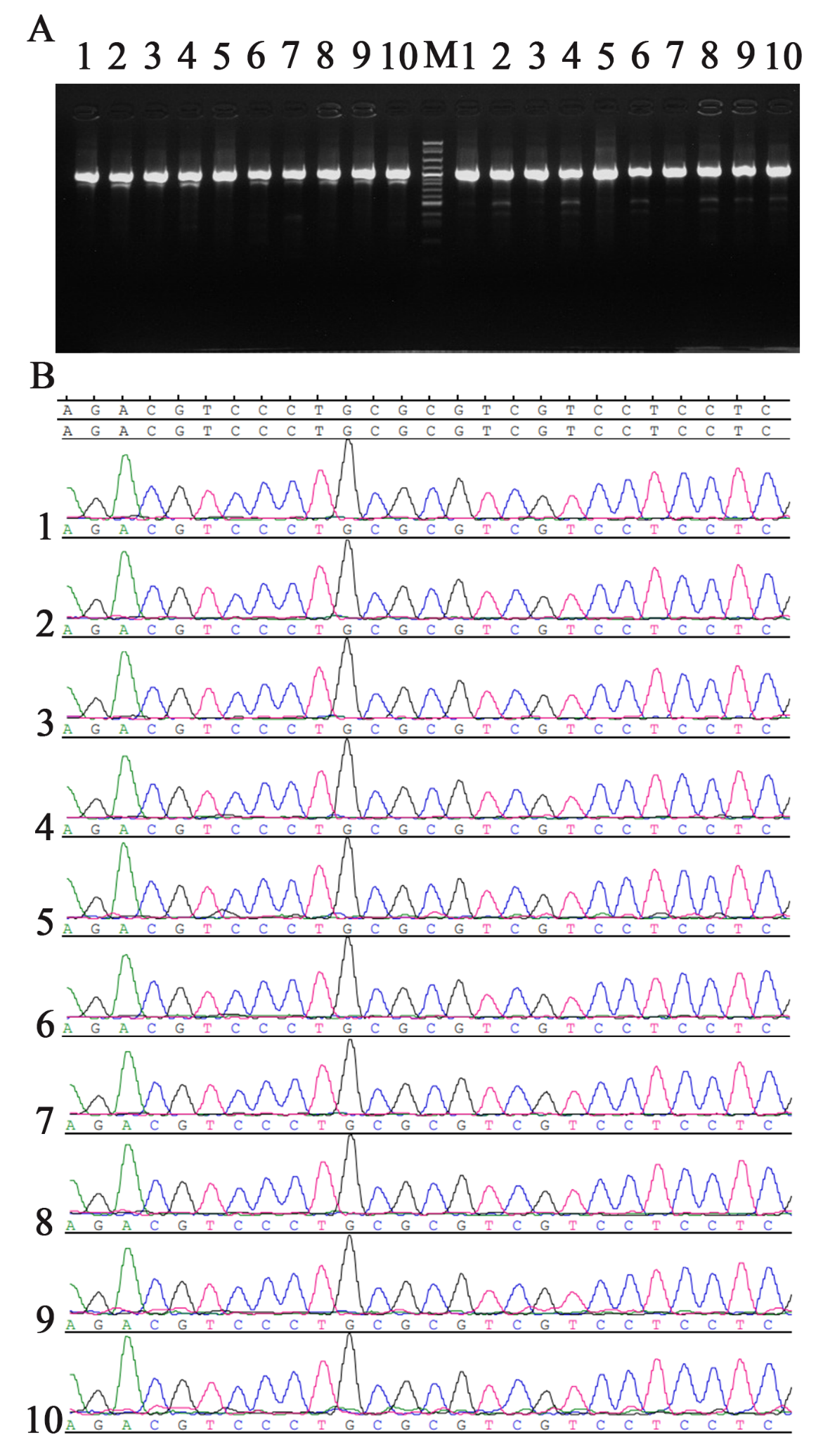
**

FIGURE 1. Singer sequencing for the detection of ATP5D gene mutation sites in male sperm. (A) The size of PCR products was detected by electrophoresis. The detection was repeated twice. The genomic DNA was extracted from 3 samples from the normal group and 7 samles from the infertile group. (B) The comparison of partial sequencing sequence results (No. 1-10 represent 10 samples respectively; M: Marker).

Supplementary Figure 2


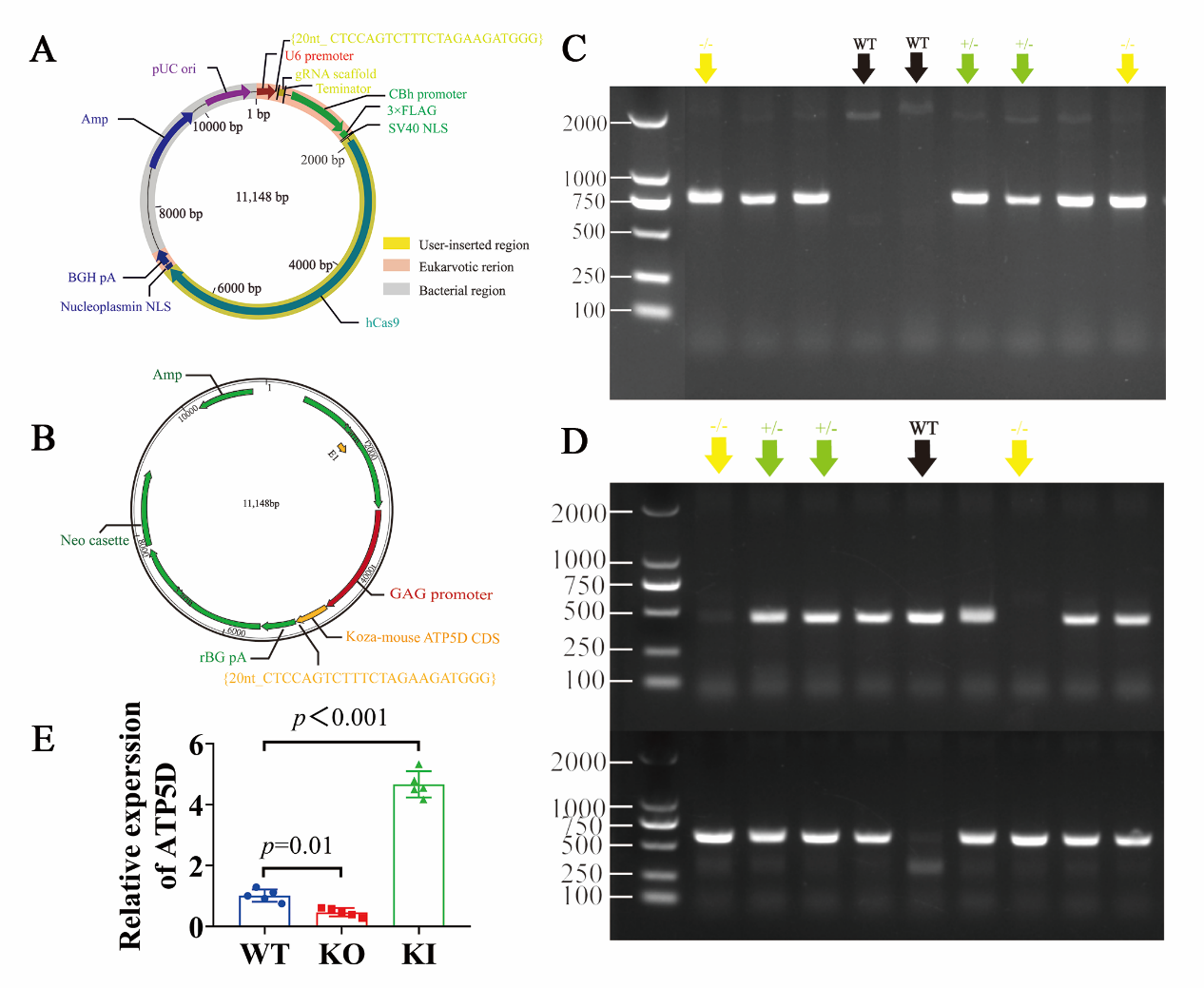
 FIGURE 2 Design for gene knockout targets and genotyping identification of *ATP5D* transgenic mice. (A, B) Gene knockout target carriers for KO and KI mice respectively. (C, D) Genotype identification for electrophoresis bands of *ATP5D* KO/KI mice. **(E)** Verification of the *ATP5D* levels in the testis by PCR. Successful construction of transgenic mice. WT vs KO, *P* = 0.01; WT vs KI, *P* < 0.0,1 (*n* = 5). All data are reported as the means ± SD.
